# Beyond BRCA: A Pilot Program to Assess and Improve Knowledge of Pharmacogenomic Testing Among Advanced Practitioners in a Breast Cancer Treatment Setting

**DOI:** 10.6004/jadpro.2016.7.4.2

**Published:** 2016-05-01

**Authors:** Samuel L. Hoffman, Robert Reid Reid Kaufman, Shannon Ferrari, Sheila Ann Alexander, Margaret Quinn Rosenzweig, Susan W. Wesmiller

**Affiliations:** 1 Doctoral Program of Nursing Practice in Family Medicine, University of Pittsburgh;; 2 University of Pittsburgh, Acute and Tertiary Care;; 3 Magee-Womens Hospital, Breast Cancer;; 4 University of Pittsburgh, Health Promotion and Development, Pittsburgh, Pennsylvania

## Abstract

To provide the best available evidence-based care to their patients, advanced practitioners (APs) must become proficient in genomic competencies and remain informed regarding the availability of pharmacogenomic tests. Databases, such as the Centers for Disease Control and Prevention’s "Genomic Testing," provide guidance about pharmacogenomic testing, but many APs are not aware of these resources. This study employed a quasi-experimental pretest/posttest design using a convenience sample of APs in a large clinical outpatient breast cancer clinic to assess the knowledge base, beliefs, attitudes, and barriers regarding pharmacogenomic testing among front-line APs and increase knowledge through a targeted educational intervention. The objectives of the educational intervention were to (1) increase knowledge of the clinical indication for testing; (2) increase collaboration among the interprofessional team; and (3) identify correctly when the plan of care should be modified based on pharmacogenomic test results to optimize patient outcomes. Responses showed that these oncology APs possess a strong foundation in genetics and support the addition of new pharmacogenomic tests to their practice.

Advanced practitioners (APs), including nurse practitioners (NPs) and physician assistants (PAs), are important members of the interprofessional clinical team caring for patients with cancer. Clinical care is increasingly complex, requiring knowledge of pathophysiology and genomics for everyday practice. This is especially true in the oncology setting, where genetic testing is an important part of standard practice (National Comprehensive Cancer Network [NCCN], 2015a). Routine testing such as Oncotype DX, the sequencing of tumor tissue to determine growth patterns and drug response, is recommended by treatment guidelines for screening (NCCN, 2014) and diagnosis (NCCN, 2015b; Robson, Storm, Weitzel, Wollins, & Offit, 2010). Pharmacogenomics is the study of how genomic variation affects an individual’s response to medications (National Institutes of Health, 2015). Pharmacogenomics helps us understand why two different people respond differently to the same medication. (Table 1 is a list of excellent pharmacogenomics resources for APs.)

**Table 1:**
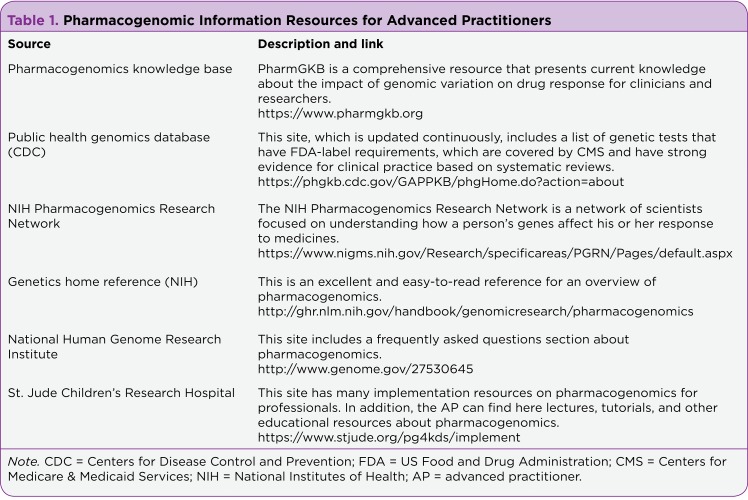
Pharmacogenomic Information Resources for Advanced Practitioners

To fully utilize the pharmacogenomic tests available, APs must know what is available as well as how to order and interpret the results. Some genetic tests have become familiar among patients and providers, such as risk assessment of the *BRCA* genes (Bellcross et al., 2011; MacNew, Rudolph, Brower, Beck, & Meister, 2010). However, pharmacogenomic tests affecting pharmacodynamics and pharmacokinetics also have important clinical utility and are less well known (Carr, Alfirevic, & Pirmohamed, 2014). Practicing APs will benefit from educational programs designed to increase their knowledge of this rapidly growing area. Resources such as databases supported by PharmGKB and the US Food and Drug Administration (FDA) are available; they catalogue the body of pharmacogenomic knowledge and rate the level of evidence for pharmacogenomic tests based on the latest information (FDA, 2015; PharmGKB, 2014).

Several professional organizations are working to provide additional support. The American Nurses Association (ANA) has developed essential genomic competencies to be integrated into standard nursing practice, which focus on assessment; identification; referral; as well as education, care, and support (ANA, 2008). These competencies were likewise endorsed by the Physician Assistant Education Association (Rackover et al., 2007). Advanced genomic competencies for nurses with graduate degrees, which emphasize ordering tests, interpreting test results, clinical management, leadership, and research in addition to the four core competencies, have also been developed (Greco, Tinley, & Seibert, 2012). Much progress has been made in the integration of core genomic competencies into curricula for health-care providers at many levels (Howington, Riddlesperger, & Cheek, 2011; Katsanis et al., 2015; Nickola, Green, Harralson, & O’Brien, 2012; Peek, 2015).

Core and advanced genomic competencies are primarily applied in academic settings, but the education of currently practicing providers is also key to clinical implementation and perhaps more difficult to achieve. There is a clear gap between published competencies and current clinical practice, demonstrating a clear need for increased education. More than half of practicing clinicians at top research hospitals were unable to identify even one indication for pharmacogenomic testing (Jerome, Solodiuk, Sethna, McHale, & Berde, 2014; Katsanis et al., 2015). In 2011, nearly all physicians surveyed believed that genetic variability can affect drug response, but only 13% had ordered a related test in the past 6 months, and only 29% had received education regarding pharmacogenomics (Pisanu et al., 2014; Stanek et al., 2012). Despite inconsistent training, physicians, APs, and other team members have a desire to learn about genomics (Katsanis et al., 2015).

Beyond a general lack of pharmacogenomic knowledge, there are other barriers to implementation, including concerns about cost, applicability, and genetics literacy (Patel, Ursan, Zueger, Cavallari, & Pickard, 2014). Costs for testing vary widely due to insurance differences. Cost concerns are best addressed by FDA and CMS (Centers for Medicare & Medicaid Services) recommendations, whereas applicability information is available through multiple databases. As part of the interprofessional team, APs need to be able to recognize those medications that may be affected by genomic factors (Howington et al., 2011; Katsanis et al., 2015).

Therefore, the primary aim of this project was to evaluate knowledge, attitudes, and use of pharmacogenomic testing among APs in the breast cancer care setting. Secondary aims included (1) identification and modification of a validated questionnaire to evaluate the knowledge base and opportunities for improvement; (2) development and implementation of an evidence-based educational intervention focused on use of clinical indications for testing to improve knowledge deficits; and (3) emphasis on a team-based approach to work through barriers to practice identified by interviews and prior to testing.

## METHODS

**Design**

This pilot study used a quasi-experimental pretest/posttest design to assess knowledge about pharmacogenomic testing before and after a targeted educational intervention.

**Participants/Setting**

Participants were recruited by convenience sampling from a women’s breast cancer center of a large tertiary hospital that employed five APs. The APs all had an advanced practice degree (MSN, MPAS), worked at least part-time in breast cancer care, and were available at three time points for data collection. All eligible providers (n = 5) agreed to be part of the project.

**Questionnaire**

A questionnaire was developed from a validated evidence-based practice assessment tool (Weng et al., 2013) for use in this study. The questionnaire was modified based on interviews with a clinical expert (a senior unit AP not participating in the project) regarding barriers to implementation, relevant practice issues, and applicable pharmacogenomic tests.

Seven questions measured self-perceived attributes key to pharmacogenomic testing on a five-point Likert scale (see top part of the Appendix on page 389). Questionnaire results for items 1 to 7 were rated according to the strength of agreement or disagreement with positive statements. High scores (4 or 5) indicated agreement, whereas low scores (1 or 2) indicated disagreement. Four true/false questions addressed clinical relevance and practice, with additional lines for written short-answer details (see bottom part of the Appendix). Two questions provided additional space for feedback and questions. Demographic information regarding training, years of practice, and additional clinical roles was gathered at pretest. Questionnaires were administered once before the educational intervention, once immediately after, and again 1 month later.

**Educational Module**

The educational module developed for this project focused on four FDA-approved pharmacogenomic tests, chosen for their relevance to an oncology unit–specific practice and offered as drug/gene pairs following the Clinical Pharmacogenetics Implementation Consortium (CPIC) convention (Table 2). Each pharmacogenomic test was paired with one medication or group of medications, and specific clinical indications and clinical impacts were reviewed. The tests used were "green" on the FDA guide (FDA, 2015), indicating full coverage by the CMS, rated evidence Level 1A by the research group PharmGKB (2014), or were otherwise clinically significant and actionable (Innocenti, 2014; Schaid et al., 2014; Spraggs et al., 2011).

**Table 2:**
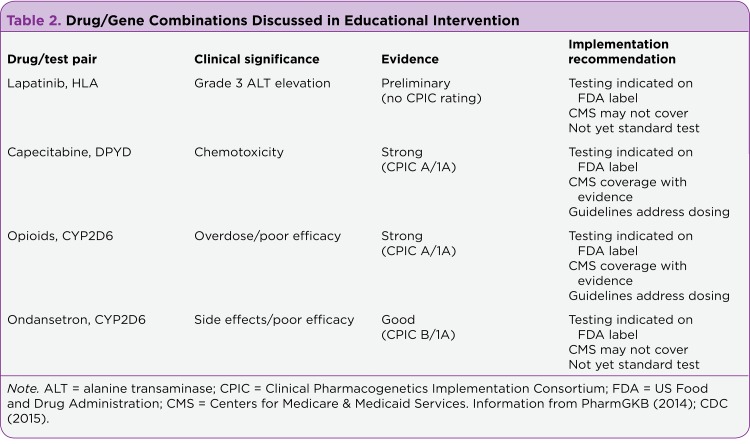
Drug/Gene Combinations Discussed in Educational Intervention

The educational intervention provided a brief overview of drug metabolism, genetic variation, and recent advances in genome sequencing. The focus was on clinical assessment of patients for indications of altered metabolism and clinical impact of testing to identify at-risk individuals (Mills, Voora, Peyser, & Haga, 2013). Advanced practitioners were advised to refer suitable cases to their collaborating physician for possible testing. Not all tests discussed are regularly covered by CMS, but each test recommended was evidence-based with potential for clinical impact. The expert AP reported that APs did not order any genomic testing independently within the facility; unit oncologists ordered the tests as indicated by family history and/or tumor presentation or the patient was referred to genetic counselors for detailed history-taking and further testing.

The intervention and questions were phrased to support team involvement as a means of working within the system’s structure. Additional resources, including links to the databases used to develop the intervention, a related module, and an article on ethical considerations of pharmacogenomic testing, were provided. The intervention is available for review upon request. The first posttest questionnaire was administered immediately after the educational intervention, and a follow-up questionnaire was administered 1 month later.

## RESULTS

Participant demographics are shown in Table 3. Participants were all female and averaged 48.2 years of age. They had an average of 23.2 years of bedside practice in various backgrounds and 7.8 years of advanced practice in oncology.

**Table 3:**
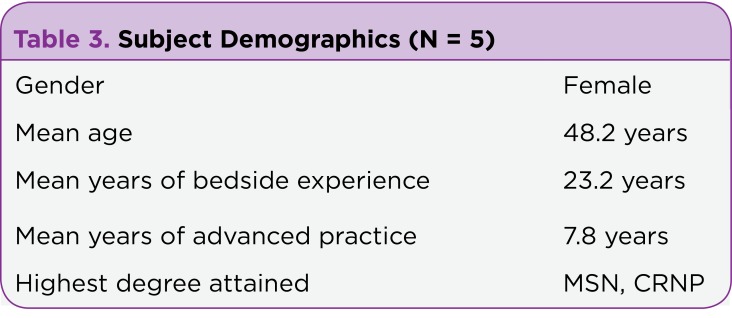
Subject Demographics (N = 5)

Pretest results indicated that the APs had prior knowledge of genetic testing to inform practice, primarily of *BRCA1*, *BRCA2*, HER/ErbB, and Oncotype DX as indicated by write-in responses to questions A, B, C, and D. Contrary to tumor-centered testing, participants reported limited clinical experience with or education regarding patients’ genomic variations affecting medication efficacy in the breast cancer setting. The pretest also identified barriers to practice, including the APs’ inability to independently order genomic testing within the institution and neutral rating regarding system support.

Study results supported that the APs were familiar with genomic testing but were unaware of pharmacogenomic options prior to the intervention (Table 4). This finding is supported and clarified by short-answer comments from pretests about which tests were indicated: "New patient requiring BRCA testing," "MammaPrint, Oncotype, Foundation One," and "Early-Stage Breast Cancer: Determine Need for Chemo." The APs agreed or strongly agreed with statements of belief in clinical utility and support for implementation.

**Table 4:**
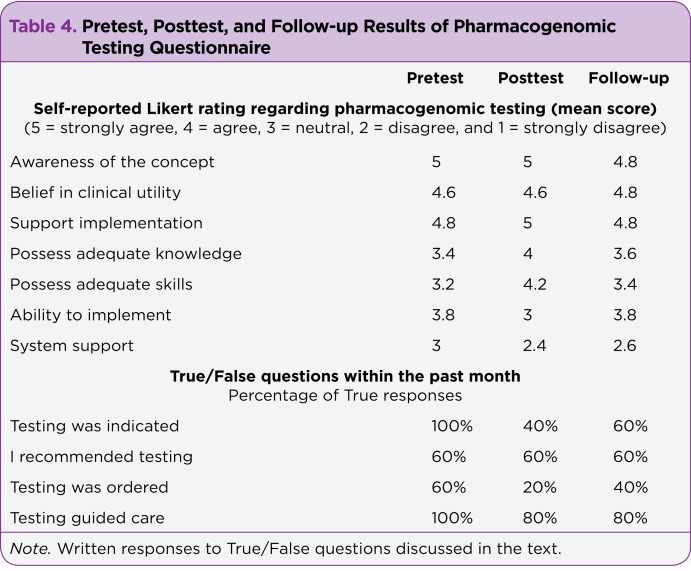
Pretest, Posttest, and Follow-up Results of Pharmacogenomic Testing Questionnaire

Participants reported less agreement with statements regarding knowledge, skills, ability to implement, and system support. Knowledge and skills improved after the educational intervention. However, the increase in agreement was not sustained at 1-month follow-up. Ability to implement testing was rated more neutrally after the intervention and returned to baseline after 1 month. System support was rated lowest of all categories across the project, with a slight drop after the intervention that was maintained at 1 month.

Indication for pharmacogenomic testing fell dramatically after the intervention from five True responses to two and three for posttest and follow-up, respectively. There was no change in recommendation of testing. Lastly, at 1 month, fewer participants reported that pharmacogenomic testing guided patient care. Two written responses are illustrative: "Mostly Oncotype, no others at this point" and "Foundation One testing, but treatment decided by MD." These items were not discussed during the intervention and were used by the physicians prior to the intervention.

## DISCUSSION

This pilot project identified preliminary evidence affirming previous research that APs working in breast cancer care were aware of cancer-specific genetic testing (Bellcross et al., 2011; MacNew et al., 2010). High initial confidence in knowledge and support dropped after the intervention due to increased awareness of pharmacogenomic testing as different from tumor typing. Participants were receptive to educational interventions regarding pharmacogenomic testing relevant to their practice, as expected (Katsanis et al., 2015). Brief educational intervention may increase APs’ knowledge and skills related to clinical testing and application. However, because APs cannot directly order pharmacogenomic tests in this setting, the knowledge was not sustained. The Institute of Medicine (IOM) recommends institutional approval for APs to practice to the full scope of their role (IOM, 2011), which includes the ordering of genomic testing and application toward a patient’s individualized care (Greco et al., 2012).

This project provides evidence that APs are able to identify appropriate opportunities for pharmacogenomic testing consistent with the ANA nursing competencies (ANA, 2008), with educational support from a provider in the DNP role. Advanced practitioners are ready to assess, identify, order tests, and manage care as per AP competencies (Greco et al., 2012) if the barrier of direct test ordering is addressed. Although scope-of-practice issues are being addressed at every level from institutions to the national stage, practice must change now. The barrier may be mitigated by improved communication with the care team. The team leaders in the unit were encouraging during this project, which reinforced the need for team-based training to support an interprofessional approach to future quality-improvement efforts (Ewing, 2015).

Both the educational intervention and questionnaire were designed to emphasize a team-based approach but did not directly involve any team members other than APs. A future study should include physicians, pharmacy, and all nursing staff. As part of the multidisciplinary health team, nurses "need to be able to recognize those medications that may be affected by genomic factors" (Howington et al., 2011; Katsanis et al., 2015).

A future project implemented in a setting where APs have the ability to order appropriate pharmacogenomic testing may have stronger results. Ideal metrics for future investigation would include institutional pharmacogenomic testing utilization and patient outcomes. Hands-on training with clearly developed guidelines for the ordering process within a given setting would facilitate more sustainable improvement in knowledge retention and application. Education of APs along with the care team will likely best address communication and access issues. APs, including DNPs, with specialized genomic knowledge are ideally suited to bring practice in line with rapidly growing bodies of evidence through targeted educational interventions.

**Figure 1:**
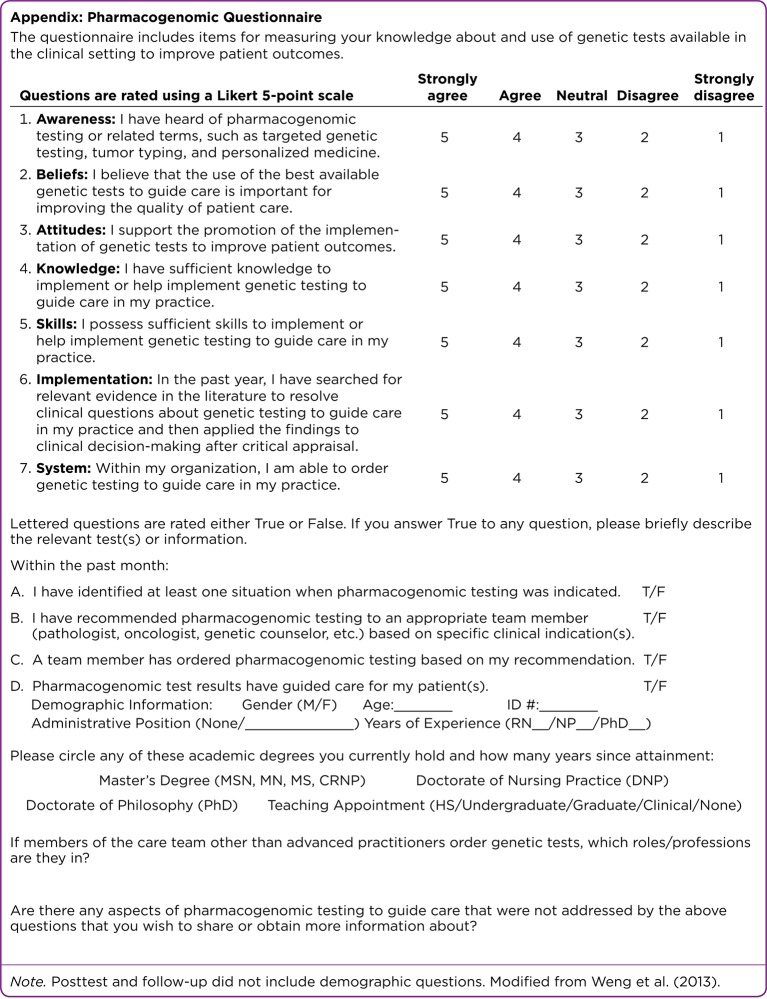
Appendix: Pharmacogenomic Questionnaire
